# Who am I with my Lewy bodies? The insula as a core region of the self-concept networks

**DOI:** 10.1186/s13195-024-01447-2

**Published:** 2024-04-19

**Authors:** Alice Tisserand, Frédéric Blanc, Mary Mondino, Candice Muller, Hélène Durand, Catherine Demuynck, Paulo Loureiro de Sousa, Alix Ravier, Léa Sanna, Anne Botzung, Nathalie Philippi

**Affiliations:** 1 University of Strasbourg and CNRS, ICube Laboratory UMR 7357 and FMTS (Fédération de Médecine Translationnelle de Strasbourg), IMIS team and IRIS platform, Strasbourg, France; 2 University Hospitals of Strasbourg,CM2R (Research and Resources Memory Centre), Geriatric Day Hospital and Neuropsychology Unit, Geriatrics Department and Neurology Service, Strasbourg, France

**Keywords:** *Self*, *Self-concept*, *Insula*, *Dementia with lewy bodies*, *Lewy bodies*, *Semantic knowledge*

## Abstract

**Background:**

Dementia with Lewy bodies (DLB) is characterized by insular atrophy, which occurs at the early stage of the disease. Damage to the insula has been associated with disorders reflecting impairments of the most fundamental components of the self, such as anosognosia, which is a frequently reported symptom in patients with Lewy bodies (LB). The purpose of this study was to investigate modifications of the self-concept (SC), another component of the self, and to identify neuroanatomical correlates, in prodromal to mild DLB.

**Methods:**

Twenty patients with prodromal to mild DLB were selected to participate in this exploratory study along with 20 healthy control subjects matched in terms of age, gender, and level of education. The Twenty Statements Test (TST) was used to assess the SC. Behavioral performances were compared between LB patients and control subjects. Three-dimensional magnetic resonance images (MRI) were acquired for all participants and correlational analyses were performed using voxel-based morphometry (VBM) in whole brain and using a mask for the insula.

**Results:**

The behavioral results on the TST showed significantly impaired performances in LB patients in comparison with control subjects (*p* < .0001). Correlational analyses using VBM revealed positive correlations between the TST and grey matter volume within insular cortex, right supplementary motor area, bilateral inferior temporal gyri, right inferior frontal gyrus, and left lingual gyrus, using a threshold of *p* = .001 uncorrected, including total intracranial volume (TIV), age, and MMSE as nuisance covariates. Additionally, correlational analysis using a mask for the insula revealed positive correlation with grey matter volume within bilateral insular cortex, using a threshold of *p* = .005.

**Conclusions:**

The behavioral results confirm the existence of SC impairments in LB patients from the prodromal stage of the disease, compared to matched healthy controls. As we expected, VBM analyses revealed involvement of the insula, among that of other brain regions, already known to be involved in other self-components. While this study is exploratory, our findings provide important insights regarding the involvement of the insula within the self, confirming the insula as a core region of the self-networks, including for high-order self-representations such as the SC.

**Supplementary Information:**

The online version contains supplementary material available at 10.1186/s13195-024-01447-2.

## Introduction

Dementia with Lewy bodies (DLB) is the second most common form of cognitive neurodegenerative disease after Alzheimer’s disease (AD), with prevalence rates of up to 5% in the elderly population and up to around 20% of all cases of dementia [1, 2]. While visual hallucinations are frequently present in patients with Lewy bodies (LB), other behavioral disturbances such as anosognosia also frequently occur (Calil et al., 2021) and could be related to an alteration of the self-consciousness. DLB is characterized by insular atrophy, occurring from the early stage of the disease [3–6], the insula being a core region for the self [7].

The insula is indeed involved in a wide variety of processes related to the self, from the most internal bodily states, such as interoception [8, 9] to high-order processes such as knowledge about oneself [10], that belong to the self-concept (SC). The SC encompasses different types of self-knowledge, such as attributes, traits, beliefs, values, social status, roles, physical characteristics or even personal preferences [11, 12]. It is made up of a body of autobiographical knowledge and refers to the way we internally represent who we are, determining what sort of person we are. The SC is closely linked to autobiographical memory, and particularly to its semanticized form, which contributes to the formation and maintenance of mental representation of the self in the present moment and across time [13–20]. Thus, behavioral and neuroimaging studies are increasingly addressing tasks related to the SC, through methods exploring personal identity. In a study investigating reflective self-referential processes, healthy subjects were asked to think intensely on how they would describe their own personality traits and physical appearance. Self-referential conditions (personality traits and physical appearance) induced activation in various cortical midline structures (CMS), including the medial prefrontal cortex (MPFC), the anterior cingulate cortex, the supplementary motor area (SMA), and the precuneus, when compared to non-self-referential conditions (i.e., thoughts about a famous person, the Danish Queen) [21]. Another study explored brain activity whilst subjects were reflecting upon their own personal qualities as compared to those of an acquaintance. For both conditions, researchers found activations in the ACC, MPFC, SMA, and superior temporal gyrus, with significantly greater activations in the self condition. Interestingly, the insula showed unique activity associated with self-reflection, highlighting a key role for the insula in self-referential processes [10].

Hence, the aim of the present study was to better understand the impact of neurodegeneration on the SC in patients with prodromal to mild DLB, and to explore the underlying structural mechanisms, with a particular focus on the link between SC and the insula. Regarding this relationship, our team has previously highlighted the existence of modifications of the SC in LB patients from the prodromal stage, in an experiment on personal tastes. By means of a questionnaire, we demonstrated that LB patients presented significant changes in tastes in both food and non-food domains, independently of the existence of anosmia or agueusia. Moreover, we found that these changes were associated with grey matter atrophy in the insula [22], suggesting a role for the insula in supporting personal tastes as self-concepts. Based on the fact that the insula is a key region for the self and given that this region is atrophied at an early stage in LB patients, we posit that impairments of the SC would be observed in LB patients, and would be correlated to insular atrophy, among other regions involved in self-concept.

## Methods

### Study population

Twenty prodromal to mild DLB patients and 20 healthy control subjects matched for age, gender and level of education (with a minimum of 9 years) were enrolled in the present study between January 2021 and February 2023. A description of the study population is presented in Table 1. Patients were recruited from the tertiary memory clinic of Strasbourg University Hospital, France, including the Geriatrics and Neurology Departments. Control subjects were recruited among friends and relatives of the patients, via the listing of controls of the local clinical investigation center and from the control group of the AlphaLewyMA cohort (http://clinicaltrials.gov/ct2/show/NCT01876459).

The LB group comprised 14 men and 6 women aged from 60 to 80 years (mean = 71.95, standard deviation [SD] = 7.99). Diagnosis of prodromal and mild DLB patients was based on core clinical features [23, 24]. Some of the patients had also benefited from biomarkers during their clinical follow-up. Indeed a dopamine transporter SPECT (DAT) scan was realized when parkinsonism was doubtful, and cerebrospinal fluid (CSF) analysis was realized when an amnestic syndrome of hippocampal type suggested a possible Alzheimer’s disease, to ensure that there was no co-pathology. Patients with prodromal DLB were defined as having mild cognitive impairment if they had a Mini-Mental State Examination (MMSE) [25] score ≥ 26, had preservation of independence as assessed by the Instrumental Activities of Daily Living [26], and fulfilled both the DSM-5 criteria of mild neurocognitive disorder [27] and McKeith’s criteria for the diagnosis of prodromal DLB [23]. Patients were defined as having mild DLB if they had an MMSE score between 20 and 25 and were diagnosed as having probable DLB according to the current DLB criteria [24]. Thus, 13 of the 20 patients were in the prodromal stage of DLB and the remaining seven were in the mild stage of the disease. Dopamine transporter SPECT (DAT) scan was available to support the diagnosis in approximately a quarter of the patients. CSF analysis was available to confirm that there was not associated Alzheimer’s disease in approximately half of the patients. All participants benefited from a classic medical examination, which notably included evaluation of the features of parkinsonism using the Unified Parkinson’s Disease Rating Scale (part 3): akinesia, rigidity, and tremor at rest (rated from 0 for no symptoms to 4 for serious symptoms). The LB group underwent further clinical examination of DLB core criteria, among which fluctuations were assessed with the Mayo Clinic Fluctuations scale [28] and the Newcastle upon Tyne Clinician Assessment of Fluctuation scale [29]. The Parkinson’s disease-associated psychotic symptoms questionnaire [30] was used to evaluate the presence of hallucinations. Rapid eye movement sleep behavior disorder (RBD) was evaluated using a sleep questionnaire on RBD [31], simplified into four questions each for the patient and the caregiver: one concerning movements during sleep, the second concerning vivid dreams and nightmares. Among the 20 LB patients, 85% presented fluctuations, 60% presented hallucinations or illusions, and 60% presented RBD. Concerning parkinsonism features, akinesia was observed in 80% of LB patients, rigidity in 70% of LB patients, and tremor at rest in only one patient (5%) (see Table 1).

Participants with history of alcohol/substance abuse, significant visual or auditory disabilities, relevant neurological of psychiatric comorbidities, or the presence of other severe or unstable medical illnesses were not enrolled in the study. Participants with an abnormal neurological examination – except for parkinsonism in the case of patients –, depression symptoms (mini-GDS [32]), , or a significant cerebral vascular burden (Modified Hachinski Ischemic Score scale > 7 [33]), were not enrolled. Participants with CSF biomarkers in favor of an Alzheimer’s disease (i.e. abnormal Aβ42/Aβ40 ratio, t-Tau, phospho-Tau181) were not enrolled. Finally, participants with claustrophobia or contraindications to MRI were not enrolled. All participants provided written informed consent for the study, in accordance with the Declaration of Helsinki, and the study was approved by the local Ethics committee (Sud Méditerranée III).

**Table 1:** Demographic and clinical characteristics of the LB group and the control group

Characteristic	LB group (*n* = 20)	Control group (*n* = 20)	Student’s t test or chi-squared test
Age (years)	71.9 (7.9)	71.2 (7.6)	*t* = 0.28, *p* = .778
Years of education	12.2 (2.2)	14.8 (4.1)	*t* = *-2*.54, *p* < .05
Sex, M/F	14/6	12/8	χ*2* = 0.44, *p* = .507
MMSE score (/30)	26.3 (2.39)	28.8 (1.0)	*t* = *-4.32, p* < .001
Handedness, R/L	19/1	20/0	χ*2* = 1.03, *p* = .311
Fluctuations (%)	17/20 (85)	N/A	
Hallucinations/illusions	12/20 (60)	N/A	
RBD	12/20 (60)	N/A	
Parkinsonism			
Akinesia (%)	16/20 (80)	N/A	
Rigidity (%)	14/20 (70)	N/A	
Tremor at rest (%)	1/20 (5)	N/A	

*LB* Lewy bodies, *M* male, *F* female, *MMSE* Mini-Mental State Examination, *R* right, *L* left, *RBD* rapid eye movement sleep behavior disorder, *N/A* not applicable

Standard deviations for age and years of education are shown in parentheses. Significant p and χ2 values are in italics

### Behavioral study

All participants underwent an evaluation of the SC. We also assessed general cognitive functioning and verbal fluency in LB patients.

### General cognitive functioning

General cognitive functioning was assessed with the MMSE, and the maximum score was 30 points.

### Verbal fluency

Verbal fluency was assessed with a task in which patients had two min to generate as many words as they could beginning with the letter P. Proper nouns and variations on words (e.g., “photograph” and “photography”) were not allowed. The score was the number of correctly generated words.

### Twenty statements test

The assessment of the SC was performed with the Twenty Statements Test (TST; [34]). The TST requires the subjects to make up to 20 statements in response to the question “Who am I?”. They were asked to write down each of the statements by completing the sentence “I am…”. To avoid any potential limitations due to impairment in generative strategies, cues indicating different possible categories of responses among five domains of identity (i.e., personal, family, social, moral, and physical) were given to the two groups of participants, as well as examples for each possible category of response. Instructions and cues were written down on the response sheet and an oral administration of the test was used for DLB patients in order to facilitate responses. For the purposes of this study, we only considered the number of responses provided by the participant, regardless of the category. Repeated responses and responses that did not answer the question of interest were not considered.

### Statistical analyses for the behavioural study

Student’s *t* test was used to compare intergroup differences between LB patients and control subjects for both demographic and behavioral quantitative data. For the behavioral data, a one-tailed *t* test was used, since we hypothesized that LB patients would have lower scores than control subjects due to impairment of the SC. We also analyzed correlations using Pearson’s test, between the TST and MMSE scores, and between the TST and verbal fluency scores.

A chi-squared test was used to compare the sex ratio between groups. A threshold of *p* < .05 was used to determine statistical significance.

### Neuroimaging study

Each participant underwent a high-resolution anatomical MRI scan within a maximum of 12 weeks after taking the TST. T1-weighted three-dimensional anatomical images were obtained using a 3T MRI scanner (Verio 32-channel Tim Siemens scanner; Siemens, Erlangen, Germany) using a volumetric magnetization-prepared rapid acquisition with gradient-echo (MPRAGE) sequence (FOV = 256 × 256 mm, image matrix = 256 × 256, slice thickness = 1 mm, repetition time = 1900 ms, echo time = 2.52 ms, flip angle = 9°).

### Voxel-based morphometry analyses

We used voxel-based morphometry (VBM) to investigate differences in grey matter volume between the healthy controls and the LB patients, and to examine the neuroanatomical correlates of the SC in both the healthy controls and LB patients. VBM analyses included image preprocessing and statistical analyses. These steps were carried out using the SPM12 software package (Wellcome Department of Imaging Neuroscience, London; http://www.fil.ion.ucl.ac.uk/) running on Matlab R2017b (MathWorks, Natick, MA, USA). Anatomical MRI images were spatially preprocessed using standard procedures [35]. All T1-weighted structural images were first segmented, bias-corrected, and spatially normalized to the Montreal Neurological Institute space using an extension of the unified segmentation procedure that includes six classes of tissue [36]. The DARTEL registration toolbox was then used to build a study-specific template and to bring into alignment all of the segmentation images. The VBM analysis was done on modulated grey matter (GM) images; that is, the GM value in each voxel was multiplied by the Jacobian determinant derived from the spatial normalization. This procedure preserves the total amount of GM from the original images. These modulated GM images were smoothed with a Gaussian kernel (full width at half maximum [FWHM]: 8 mm). Between-group voxel-based comparisons were displayed after correcting for multiple comparisons with false discovery rate (FDR; *p* < .05). To map the regions of atrophy related to disorders of the SC, we tested the correlation between the GM volume at a voxel level and the total score on the TST using the general linear model. Correlation analysis between behavioral data and GM volume was performed using a threshold of *p* = .001 uncorrected, including total intracranial volume (TIV) and age as nuisance covariates. MMSE was also considered as an additional covariate to investigate the potential impact of disease severity. A cluster spatial extent of 50 voxels was used to avoid irrelevant and isolated detections. The software Xjview (http://www.alivelearn.net/xjview8/) allowed us to characterize each cluster.

Moreover, to ensure that finding the insula was not a spurious result, when considering the number of covariates and that we did not use correction for multiple comparison, we completed the VBM analyses in whole brain, with a VBM analysis using a mask for the insula. The GM images were smoothed at 4 mm to increase specificity in order to discriminate true effect from random noise. We used a threshold of *p* < .005, including the same covariates as for the whole brain analyses.

## Results

### Behavioral study

The behavioral results indicated that the LB group had significantly decreased performances for the total score on the TST compared to the healthy controls (mean 9.7, SD 4.5 vs. 19.05, SD 3.36, *t* = 7.38, *p* < .0001). We completed correlational analyses in the LB group by examining correlations between the TST and MMSE scores, which revealed significant correlations (*r* = .49, *p* < .05). We also analyzed correlations between the TST and verbal fluency scores, which showed significant correlations (*r =* .55, *p* < .05).

### Neuroimaging study

Voxel-based analysis comparing grey matter volume in LB patients versus healthy controls is presented in supplementary material. Analysis included TIV and age as nuisance covariates, and revealed patterns of cerebral atrophy typically reported in LB patients [4], with grey matter volume in insular, temporal, occipital, frontal, cingular cortices and to a lesser extent parietal cortex (*p* < .05, FDR corrected), in LB patients compared to healthy controls.

**Fig. 1:**
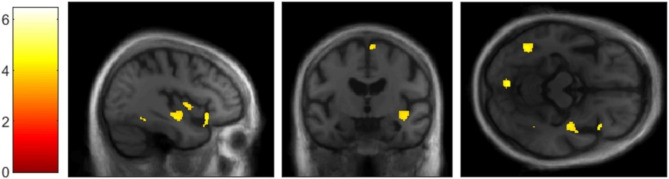
VBM analyses for self-concept in the Lewy bodies group.Grey matter volumes within right insular cortex positively correlated with the total score on the TST questionnaire (self-concept), using a threshold of *p* = .001 uncorrected, including age, TIV and MMSE score as nuisance covariates, *k* = 50

**Fig. 2:**
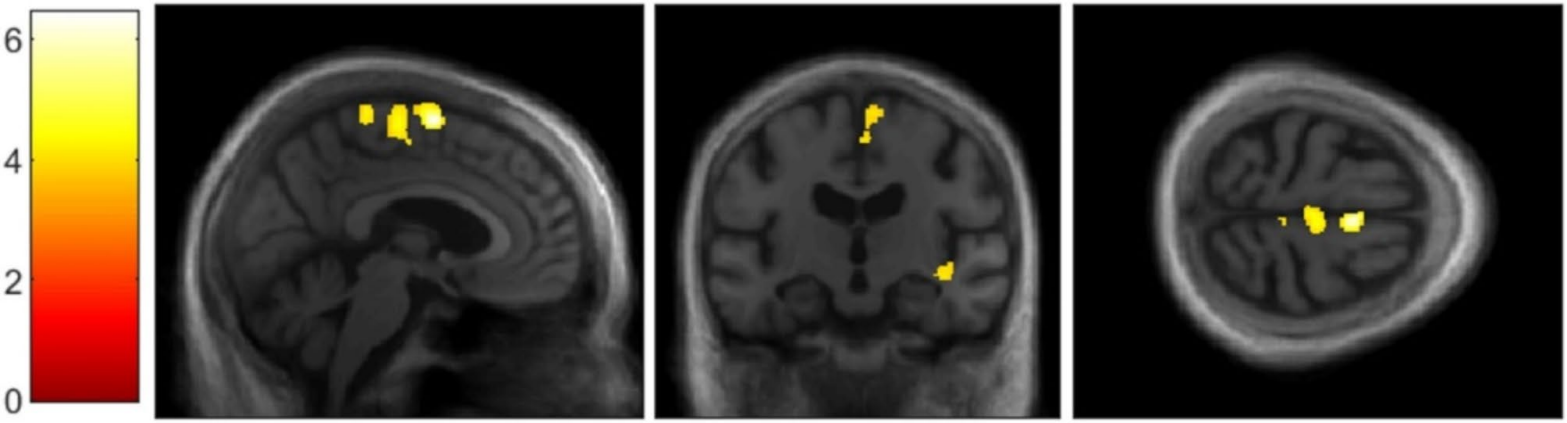
VBM analyses for self-concept in the Lewy bodies group.Grey matter volumes within right supplementary motor area positively correlated with the total score on the TST questionnaire, using a threshold of *p* = .001 uncorrected, including age, TIV, and MMSE score as nuisance covariates, *k* = 50

**Fig. 3:**
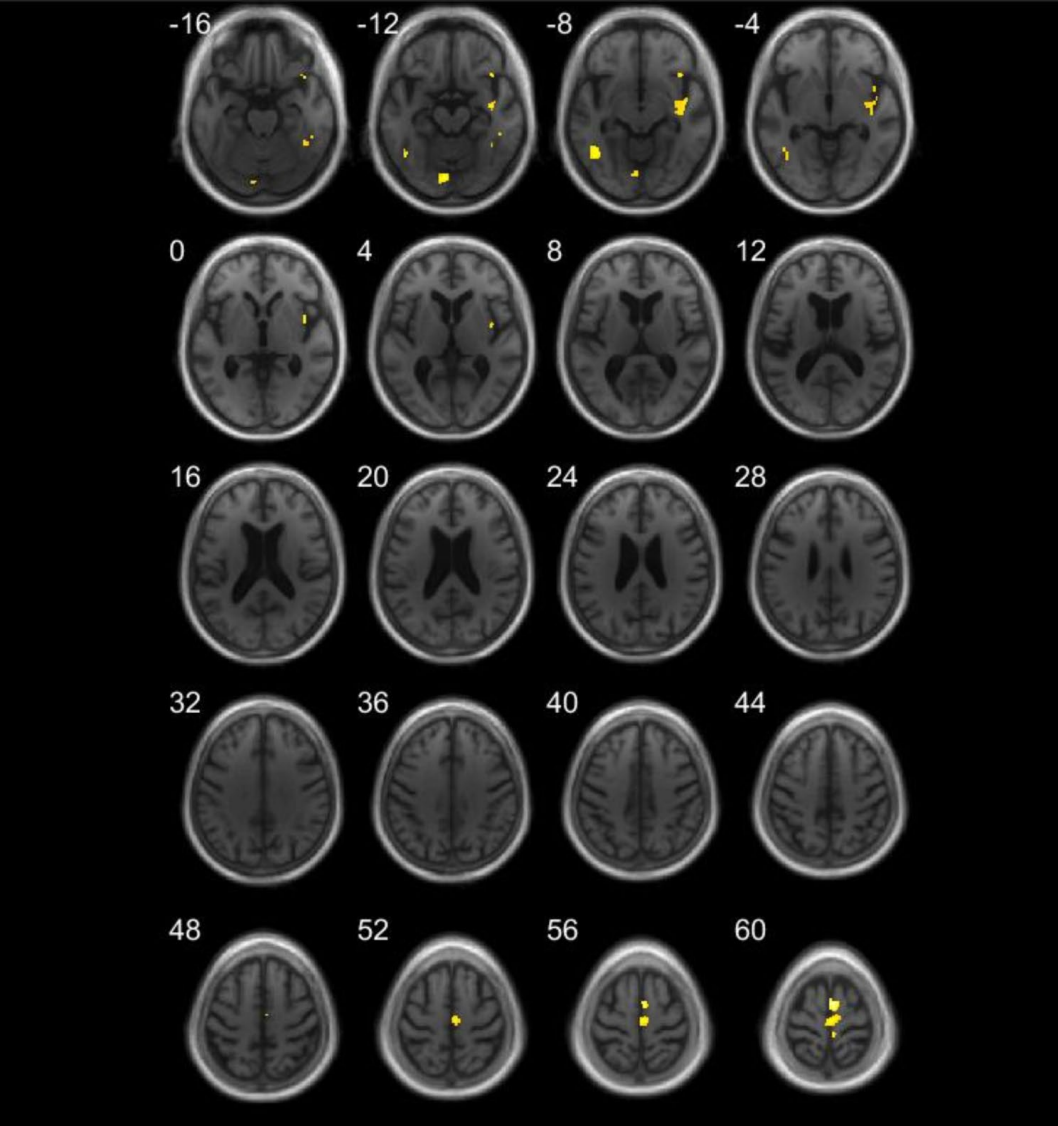
VBM analyses for self-concept in the Lewy bodies group. Grey matter volumes within right insular cortex, right SMA, bilateral inferior temporal gyri, and left lingual gyrus positively correlated with the total score on the TST questionnaire, using a threshold of *p* = .001 uncorrected, including age, TIV, and MMSE score as nuisance covariates, *k* = 50

**Fig. 4:**
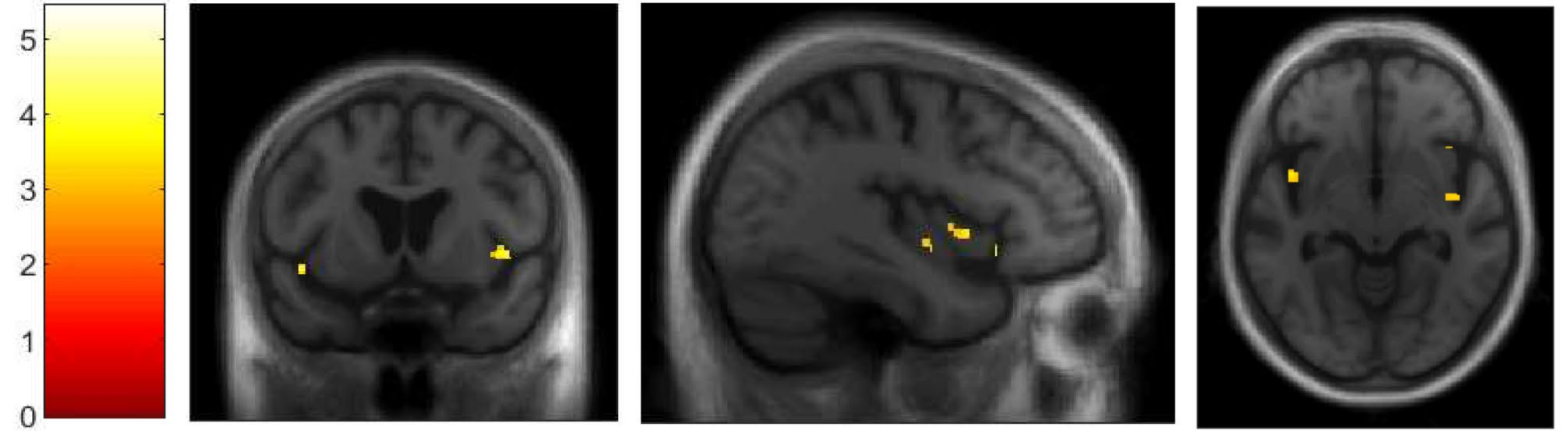
VBM analyses for self-concept in Lewy bodies group, using a mask for the insula.Grey matter volumes within bilateral insular cortex, using a threshold of *p* = .005 uncorrected, including age, TIV, and MMSE score as nuisance covariates

In the LB group, correlational analysis for the SC revealed positive correlations with GM volume within right insular cortex (see Fig. 1), right SMA (see Fig. 2), right inferior frontal gyrus, left lingual gyrus and bilateral inferior temporal gyri (see Fig. 3), using a threshold of *p* = .001 uncorrected, including TIV, age, and MMSE as nuisance covariates, with a minimum cluster size of *k* = 50 (Table 2). Correlational analysis for the SC, using a mask for the insula revealed positive correlation with GM volume within bilateral insular cortex (see Fig. 4), using a threshold of *p* = .005 uncorrected, including TIV, age, and MMSE as nuisance covariates. The correlational analyses did not reveal any correlation in the healthy controls.

**Table 2:** VBM results for the total score on the Twenty Statements Test in the Lewy bodies group

VBM	Side	BA	k	X	y	z	T
Posterior insula	R	13/21/22	91/333	45	-4.5	-6	3.92
Anterior insula	R	47	35/64	39	19.5	-12	4.3
Supplementary motor area	R	6	506/599	3	-3	58.5	6.43
Fusiform gyrus	R	37	133/185	48	-45	-22.5	4.7
Inferior frontal gyrus	R	47	64/64	40.5	18	-16.5	4.29
Lingual gyrus	L	18	155/160	-7.5	-81	-13.5	4.55
Inferior temporal gyrus	L	37	107/207	-43.5	-64.5	-3	3.92

*L* left, *R* right, *BA* Brodmann area, *k* cluster size in voxels (specific region’s volume/cluster’s global volume), x, y, z Talairach coordinates, T T-value

## Discussion

This study is the first to examine SC in LB patients by using a standardized measure. In summary, the findings indicate the existence of significant impairments of the SC in LB patients from the early stage of the disease, compared to age-matched healthy controls. Concordant with our hypothesis, imaging analyses confirmed the involvement of insular cortex atrophy in SC impairments, among other brain regions such as the SMA. These results bring new insight into the role of the insular cortex in the conception of oneself, among other brain regions more widely associated with classic self-networks.

We focused in particular on the role of the insula in the SC, with the hypothesis that insular atrophy could explain alterations of the self in LB patients. The insular cortex is commonly associated with interoceptive awareness [37] and socio-emotional processes [9]. It has also been associated with self-reflection upon personal qualities [10] and with self-esteem, two abilities that are related to the SC [38]. Regarding LB patients, our study is the first to highlight a diminished SC in relation to the insular atrophy that occurs early in the course of the disease [3–6]. SC impairments have already been linked to the atrophy of this structure in DLB, regarding modifications of personal tastes [22]. However, from the perspective of cognitive models of the self, personal preferences also imply the subjective sense of self, since liking or disliking an object is inherent to the subject in the present moment. In our study, to measure the SC in isolation from the subjective sense of self, we asked the participants to simply answer the question “who am I?”, which refers to the overall representation of oneself. In the present study, we found that both the posterior and anterior part of the insular cortex are involved in SC. The insular cortex is known to be topographically organized with a degree of complexity increasing along a posterior-anterior axis [8]. Whereas the posterior insular cortex is reported to be involved in processing a variety of bodily sensations and also in higher order somatosensory function, such as the subjective sense of body ownership and agency [39–42], the anterior insular cortex contains the representations of bodily reactions in response to affective feeling states (i.e., somatic markers) [43], sustains visual self-recognition [44, 45], and is involved in metacognitive processes [46]. Considering the involvement of the posterior insular cortex, our results suggest that this region supports conceptual aspects of the self, in addition to the most elementary aspects of subjective sense of self. One could hypothesize that the alteration of the subjective sense of self could lead to impairments of the other high order self-components such as SC, through a global collapse of the self. We posit that such damage could disrupt the feeling of what belongs to the self or to the non-self, thus making difficult the access to specific knowledge about the self. Nevertheless, since we did not focus on the subjective sense of self in the present work, our study does not confirm this hypothesis. However, it is concordant with the fact that hypoperfusion in the insula has been associated with Capgras syndrome in DLB [47], which consists of the loss of familiarity toward relatives, who are logically integrated by extension in one’s self. Concerning the anterior insular cortex, based on its role in self representations [10, 22, 38] and its involvement in metacognition [48], it appears as a main region in the conceptualization of the self. Thus, dysfunction of the anterior insular cortex could disturb the conception we have built of who we are and therefore reduce the ability to answer the question “who am I ?”.

Our second main finding concerns the relationship between CMS and the SC. In line with previous studies [49–51], we found that self-referential processes are associated with medial cortical regions, notably the right SMA. The SMA is not classically associated with the CMS as described by Georg Northoff. This area is responsible for planning of complex movements [52] and would sustain the sense of agency [53]. The SMA is well known to entail mirror neurons [54], described in the context of social cognition, rather than in self-cognition. Indeed, mirror neurons are visuomotor neurons discharging when we are executing a motor act or when we observe someone executing a similar motor act [55, 56]. Based on similar mechanisms, the mirror neuron system fires for feelings, such as empathy [57], and is considered to underlie theory of mind [58], which is the ability to infer and reason about another person’s mental states [59]. Theory of mind would be based on areas involved in the self brain network, such as the MPFC and the precuneus, and could be supported by representations of the self [60, 61]. Yet, the self-component associated with the SMA would rather pertain to the domain of the subjective sense of self, notably the sense of agency (e.g., when initiating a movement), whereas our study focuses on the conceptual representations of the self. From the perspective of embodied cognition, we can hypothesize that such a region able to simulate our motricity could also support high-order representations of the physical self, actually pertaining to the SC. These findings illustrate the concept of embodied cognition, as described by Binder and Desai for general semantic memory [62]. Indeed, the authors demonstrated that modality-specific systems are recruited when integrating supramodal representations, depending on the categories of representation (e.g. action words referring to face, arm, or leg activate frontocentral motor areas according to a somatotopic organization [63]). Concordant with this hypothesis, it has been demonstrated that mirror-neuron areas are engaged in tasks of self-face recognition [64] and would enable physical other-to-self mapping [65]. Thus, these specific neurons could play a major role in identification and representation of the physical self, which is part of the SC.

Aside from the involvement of insula and CMS, we also found correlations between impairments of the SC and diminished grey matter volume in bilateral inferior temporal gyri – including right fusiform gyrus –, left lingual gyrus and right inferior frontal gyrus. Interestingly, it has been proposed that the inferior temporal gyri, notably the anterior part, might play a key role in semantic memory [66]. In our study, we found correlations in the posterior part, in LB patients, which could be associated with the personal aspects of semantic knowledge. More specifically, the right fusiform gyrus is known to be specialized in face perception [67, 68]. Additionally, Kircher et al. found involvement of the left fusiform gyrus in personality-traits describing the self and perception of own face [45], suggesting a strong involvement of this region in self-processing. Regarding the lingual gyrus, it is a region essential for visual perception [69] and visual memory [70]. Likewise, the lingual gyrus plays a central role in divergent thinking [71], which refers to the processes allowing one to generate creative ideas by exploring different possibilities. We could speculate that divergent thinking is involved in the creation of oneself prototype. Interestingly, we found diminished grey matter volume in the right inferior frontal gyrus, which has been related to verbal fluency [72]. Yet, the TST score and verbal fluency were positively correlated in our study and could reflect research strategies in memory. Another ability that is necessary to describe one’s own SC is introspection, which has been associated with inferior frontal gyrus by means of multiband functional MRI during structured real-time conversations, in which the participant verbalized introspected thought and feeling [73]. Impairments in self-introspection could explain a poorer SC, and even be linked to impairments in introspection of consciousness, related to anosognosia. Indeed, introspection is closely linked to metacognition which is part of the subjective sense of self [18]. Subjective sense of self and SC are two interrelated concepts and distinguishing between them represents a real experimental challenge.

Overall, our study has some limitations. First, the sample sizes were relatively small (*N* = 20 in each group) with a high number of covariates and the results for the VBM analyses were uncorrected. These preliminary results should therefore be interpreted carefully and be confirmed with additional studies, involving a larger cohort of DLB patients. Moreover, comparing the SC across different clinical populations (e.g. fronto-temporal lobar degeneration, AD) at the same stage would highlight the impact of different neurodegenerative processes on the SC. Second, it would have been useful to assess and integrate other components of the self, such as subjective sense of self and autobiographical semantic memory, as additional covariates, in order to better isolate the SC. Therefore, our future work aims to investigate the relationship between the subjective sense of self, the SC, and autobiographical memory in both DLB and AD, with a special focus on the insula’s role within the different components.

## Conclusions

Our work on the SC in DLB has both clinical and theoretical interest. First, it is the first study to demonstrate the existence of SC impairments in LB patients. Concordant with our hypothesis, these difficulties are related to insular atrophy, which occurs early in the course of the disease. Moreover, our study confirms the involvement of the SMA in the self, and notably suggest a role for mirror neurons. Finally, we found that bilateral inferior temporal gyri, right inferior frontal gyrus and left lingual gyrus also play a role in the SC. This finding brings new insights regarding the implication of the insula in the different aspects of the self. Our results show that the SC relies on the insula and other brain regions involved in the other components of the self, such as the subjective sense of self. These findings suggest that conceptual representations of the self could arise from the primary phenomenological levels.

### Electronic supplementary material

Below is the link to the electronic supplementary material.

Supplementary Material 1

## Data Availability

The datasets used and/or analyzed during the current study are available from the corresponding author on reasonable request.

## Abbreviations

DLB: Dementia with Lewy bodies
LB: Lewy bodies
SC: Self-concept
MRI: Magnetic resonance imaging
AD: Alzheimer’s disease
CMS: Cortical midline structures
SMA: Supplementary motor area
SD: Standard deviation
DAT: Dopamine transporter SPECT
CSF: Cerebrospinal fluid
MMSE: Mini-Mental State Examination
RBD: Rapid eye movement sleep behavior disorder
TST: Twenty Statements Test
VBM: Voxel-based morphometry
GM: Grey matter
FDR: False discovery rate
TIV: Total intracranial volume
