# Supplementary material for: Who am I with my Lewy bodies? The insula as a core region of the self-concept networks
Source: Alzheimers Res Ther. 2024 Apr 19;16:85. doi: 10.1186/s13195-024-01447-2 (PMC11027417; doi:10.1186/s13195-024-01447-2)
Supplement: Supplementary file 1 — Supplementary Material 1 [file 13195_2024_1447_MOESM1_ESM.docx]

**Supplementary material**


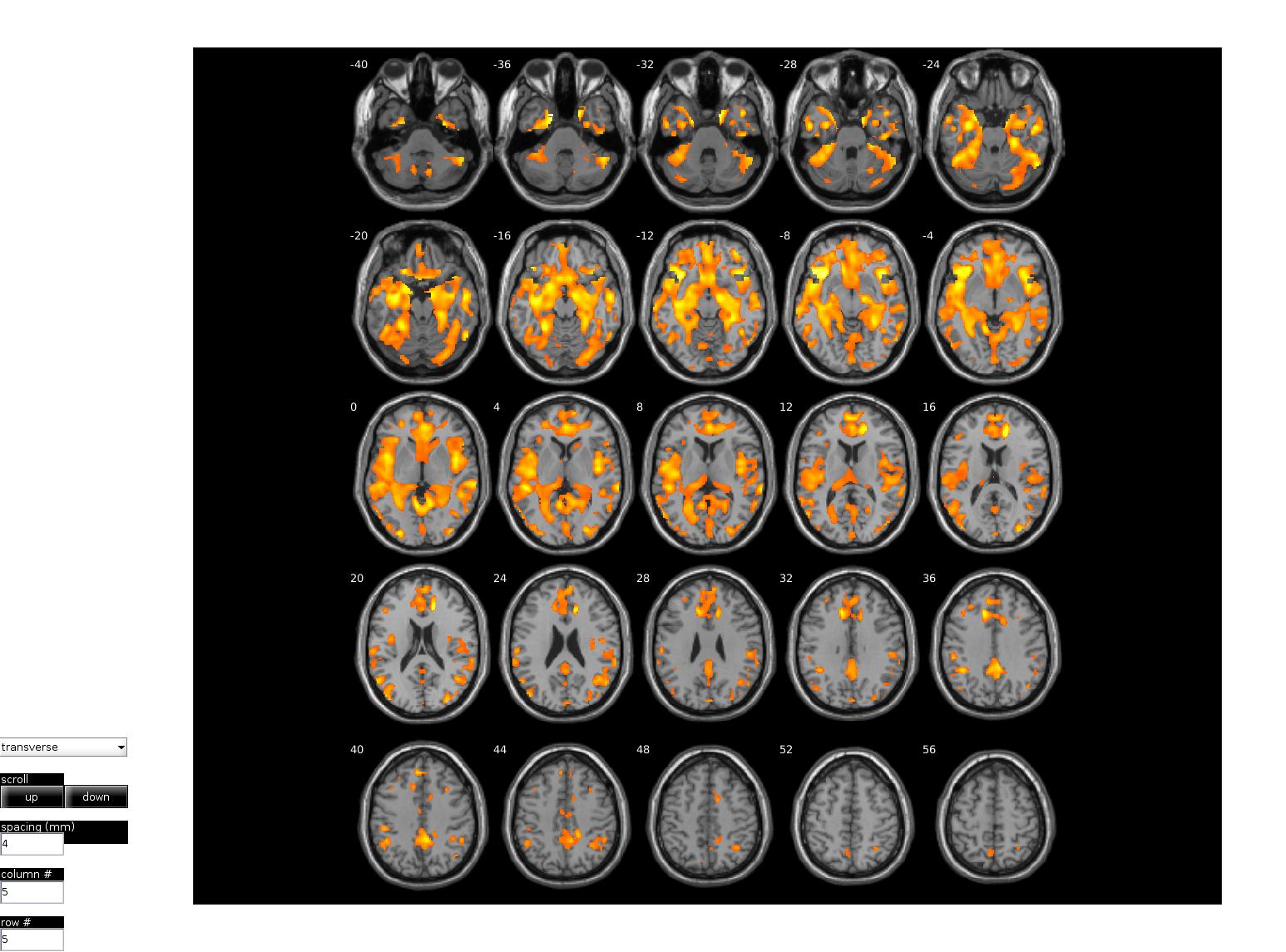


VBM analyses in LB patients compared to healthy controls

Cortical thinning involving insular, temporal, occipital, frontal and to a lesser extent parietal regions in LB patients compared to healthy controls, including TIV and age as nuisance covariates (p < .05, FDR corrected)
